# Chitosan/Hyaluronan and Alginate-Nanohydroxyapatite Biphasic Scaffold as a Promising Matrix for Osteoarthritis Disorders

**DOI:** 10.34172/apb.2024.005

**Published:** 2023-07-22

**Authors:** Seyed Abdolvahab Banihashemian, Soheila Zamanlui Benisi, Simzar Hosseinzadeh, Shahrokh Shojaei, Hojjat Allah Abbaszadeh

**Affiliations:** ^1^Advanced Medical Sciences and Technologies Department, Faculty of Biomedical Engineering, Central Tehran Branch Islamic Azad University, Tehran, Iran; ^2^Stem Cell Research Center, Tissue Engineering and Regenerative Medicine Institute, Tehran Central Branch, Islamic Azad University, Tehran, Iran; ^3^Medical Nanotechnology and Tissue Engineering Research Center, Shahid Beheshti University of Medical Sciences, Tehran, Iran; ^4^School of Advanced Technologies in Medicine, Shahid Beheshti University of Medical Sciences, Tehran, Iran; ^5^Islamic Azad University Central Tehran Branch, Department of Biomedical Engineering, Tehran, Iran; ^6^Laser Application in Medical Sciences Research Center, Shahid Beheshti University of Medical Sciences, Tehran, Iran; ^7^Hearing Disorders Research Center, Loghman Hakim Hospital, Shahid Beheshti University of Medical Sciences, Tehran, Iran

**Keywords:** Osteoarthritis, Hydrogel, Biphasic scaffold, Polymer, Mineral, Cartilage and Bone

## Abstract

**Purpose:**

Regenerative medicine offers new techniques for osteoarthritis (OA) disorders, especially while considering simultaneous chondral and subchondral regenerations.

**Methods:**

Chitosan and hyaluronan were chemically bound as the chondral phase and the osteogenic layer was prepared with alginate and nano-hydroxyapatite (nHAP). These scaffolds were fixed by fibrin glue as a biphasic scaffold and then examined.

**Results:**

Scanning electron microscopy (SEM) confirmed the porosity of 61.45±4.51 and 44.145±2.81 % for the subchondral and chondral layers, respectively. The composition analysis by energy dispersive X-ray (EDAX) indicated the various elements of both hydrogels. Also, their mechanical properties indicated that the highest modulus and resistance values corresponded to the biphasic hydrogel as 108.33±5.56 and 721.135±8.21 kPa, despite the same strain value as other groups. Their individual examinations demonstrated the proteoglycan synthesis of the chondral layer and also, the alkaline phosphatase (ALP) activity of the subchondral layer as 13.3±2.2 ng. After 21 days, the cells showed a mineralized surface and a polygonal phenotype, confirming their commitment to bone and cartilage tissues, respectively. Immunostaining of collagen I and II represented greater extracellular matrix (ECM) secretion in the biphasic composite group due to the paracrine effect of the two cell types on each other.

**Conclusion:**

For the first time, the ability of this biphasic scaffold to regenerate both tissue types was evaluated and the results showed satisfactory cellular commitment to bone and cartilage tissues. Thus, this scaffold can be considered a new strategy for the preparation of implants for OA.

## Introduction

 One of the life-threatening diseases of older people is osteoarthritis (OA) due to its complications such as joint pain and deformity that can lead to functional disability.^1^ A recent therapeutic strategy is surgical methods, sometimes combined with pharmacological interventions, especially to reduce pain. In particular, joint replacements via surgeries can fairly relieve normal mechanical motions, and thereby, a complete cure of OA is actually impossible. New therapies such as tissue engineering can address these unhealthy conditions and improve the quality of life in the population with osteochondral defects. However, this field tries to offer an adequate strategy with new materials and cell sources.^2^ Biphasic scaffolds with a two-layer construction could restore osteochondral function without the need for invasive surgery. Actually, these scaffolds are designed as a 3D model with appropriate elasticity to mimic the physiology of this tissue better. In one study, a biphasic hydrogel consisted of alginate with methacrylated chondroitin sulfate (CSMA) and cryloyl chloride-poly(ε-caprolactone)-poly (ethylene glycol)-poly(ε-caprolactone)-acryloyl chloride (PECDA). The bilayer scaffold was implanted in rabbits and the results confirmed enhanced repair of osteochondral injuries.^3^ A further investigation was performed using bioactive glass and glycol chitosan - alginate. The observations were consistent with chondral repairs and the bioglass layer showed the best osteogenic regeneration.^4^ In an interesting examination, polyacrylamide cross-linked with hydroxyapatite (nHAP) was used as the subchondral layer and when this polymer was modified with chromium acetate, it was considered as the articular phase. The corresponding study approved favored osteochondral differentiation by evaluating gene expression and cell staining methods.^5^ By carefully looking at polymers for the chondral and subchondral layers, it was found that some polymers with biochemical properties can induce cells into these tissues without the need for chemical factors. Hyaluronic acid (HA) and chitosan can form a scaffold with chondrogenic function and mechanical resistance originating from HA and chitosan, respectively, and guide cells such as human mesenchymal stem cells (hMSCs) to the chondral lineage. Indeed, the analysis confirmed their chondral regeneration as a function of HA. This hybrid scaffold caused hMSCs to express cartilage marker genes.^6^ Its main mechanism in chondral promotion depends on the positive regulation of CD44 and transforming growth factor beta receptor II (TGF-βRII).^7^ Another group found that human embryonic stem cells (hESCs) which were cultured within an HA-based scaffold, had longer-term of regenerative capacity against osteochondral damages.^8^ Another interesting property of this polymer is its specific anti-inflammatory feature, which is related to its ability to interact with cell receptors.^7^ The subchondral phase of a biphasic scaffold should have a natural potency to induce cell fate for osteogenic lineage. Alginate is one of the polymers that could be easily cross-linked only by its incubation with calcium ions, and even the degree of porosity is adjusted by the concentration of this ion.^9^ Recent reports confirm that this polymer has the potential to trigger bone regeneration and also vascularization of this tissue.^10^ However, its mechanical properties are weak and as a soft polymer, it has low cell interactions.^11^ Another characteristic of this polymer which should be highlighted, is its osteoinductive capacity, and regarding this, the corresponding polymer is not comparable to the biochemical factors such as TGF-β and bone morphogenetic protein 2 (BMP-2). To progress alginate deficiencies, nano-hydroxyapatite (nHAP) should be added to reinforce the scaffold of the osteogenic phase, due to the similarities between this nanoparticle and the specific bone microenvironment.^12^ In this way, the similarities relate to the fact that nHAP is the major mineral of a healthy bone tissue.^13^ The true cause of the osteoinductive properties of this nanoparticle, is relevant to its beneficial effect on the expression of genes involved in osteogenic differentiation such as alkaline phosphatase (ALP).^14^ Moreover, nHAP converts the soft alginate hydrogel into a scaffold with a higher degree of rigidity and this property facilitates cell attachment and mechanical integration between cells and scaffolds. On the other hand, a suitable cell source plays a crucial role in the regeneration process. Regarding this, chondrocytes and hMSCs were chosen as chondral and subchondral sources respectively in the present study.

 For the first time, in the present study, two groups of hydrogels including chitosan-HA and alginate-nHAP were prepared and their abilities for cartilage and bone regeneration were verified separately. It should be added that the porosity of the hydrogels was adjusted to have the networks with normal diffusion of nutrients and gases. Additionally, mechanical evaluations were done to ensure their functional durability and after cell seeding, their regenerative abilities were evaluated in dual and distinct phases through spectroscopy and staining methods.

## Methods and Materials

###  Preparation of N-carboxyethyl chitosan (CEC)

 The method of CEC synthesis was done in accordance with other reports,^15^ although some brief modifications were necessary. To modify the amine side groups of chitosan (Sigma, high molecular weight) with carboxyethyl substitutes, this polymer was dissolved in acrylic acid (Merck) solution (4%) at a concentration of 2%. The mixture was stirred at 50 °C for two overnights and the pH was then adjusted to 10-12 by NaOH (Sigma) and the residual material was dialyzed by dialysis tubes (MW of 12-14 kDa) in distilled water (Merck) for 3 days. Finally, the resulting polymer was lyophilized by employing a freeze-drying apparatus (Heraeus, Germany).

###  Preparation of aldehyde hyaluronic acid (AHA) 

 Hyaluronic acid polymer (Sigma, 8-15 kDa) was converted to the aldehyde form using sodium periodate (Sigma, 3%) at a concentration of 1.5%.^16^ The solution was mixed for 24 hours and diethylene glycol (Sigma) was added at an equivalent value to inhibit the oxidation reaction. The modified polymer was dialyzed against water for 3 days and finally, the material was lyophilized.

###  Preparation of CEC/AHA hydrogel

 The resulting CEC and AHA were separately dissolved in water in respective concentrations of 20% and 5% and then, their homogeneous media were mixed together in the ratio of 1:2 and stored at 8-10 °C for 24 hours. The corresponding hydrogel was used for the following tests.

###  Preparation of alginate/ nanohydroxyl-apatite ( nHAP ) hydrogel

 The osteoinductive phase of the biphasic hydrogel was made by using alginate (Sigma) and nHAP (Sigma, 200 nm). For the process, the polymer was dissolved in water at a concentration of 5% and the nanoparticles were added with the final percent of 4%. Eventually, 18% calcium solution (Sigma) was added and the hydrogel was created immediately.

###  Preparation of biphasic hydrogel

**Figure 1:**
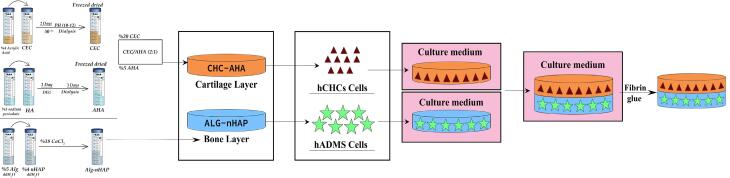
The schematic view of the bilayer scaffold synthesis and co-culture cell seeding strategy. a) The solutions of 4% CEC and %5 AHA were individually prepared and mixed with each other to develop the chondral layer. Then, the corresponding scaffold was cultured by hCHCs, b) The subchondral layer was prepared mixing of 5% alginate and 4% nHAP and then, cultured by hADMS cells and c) The chondral and subchondral layers were assembled by using fibrin glue and then, the scaffolds were adhered to fabricate the biphasic scaffold. CEC, AHA, hCHC cells and hADMS cells are the abbreviations of N-Carboxyethyl Chitosan, aldehyde hyaluronic acid, human chondrocytes and human adipose derived mesenchymal stem cells, respectively

 Fibrin glue (Hangzhou Pull Biotech Co. Ltd, China) was used to join both types of biphasic hydrogels, including the chondral and subchondral layers. Regarding this, 500 µL of each hydrogel group touched each other once the sealant (fibrin glue), was applied. Both constructs were immediately conjugated after applying fibrin glue and the samples were placed in cell culture plates for the following assays. Figure 1 shows the schematic process of the biphasic scaffold preparation.

###  Characterization of hydrogels by scanning electron microscopy (SEM) 

 The fabricated scaffolds were examined by SEM/EDAX (Seron Technologies - AIS2100 model, Korea). For this assay, the hydrogel samples after their freeze-drying process, were coated with gold by ion sputter (JFC-1100, JEOL, Japan) and then, their morphology and elemental compositions were examined by SEM and energy dispersive X-ray (EDAX). Furthermore, cell morphology after the cell seeding on these scaffolds including alginate type in the presence and absence of nHAP and also, CEC in combination with and without AHA were investigated by SEM. For this evaluation, after 21 days, the cells were fixed with glutaraldehyde (Merck, 2%) for 40 minutes at room temperature. The cells were then dehydrated through a 50-100% serial dilution of ethanol (Merck). Each incubation with ethanol, was considered 20 minutes at room temperature. The specimens were coated with gold and the shape of cells on the scaffolds was studied by SEM.

###  Measurement of hydrogel porosity by ImageJ software 

 The SEM images of the hydrogel samples were investigated by ImageJ software (LOCI, University of Wisconsin) to determine their porosity values. After installing ImageJ, the SEM image datasets were run and converted to binary types and their thresholds were adjusted. In order to generate the images with pores, the pixel units and also, the shape of the pores were plotted in their associated complements and finally, the position of the pores and their surface area were obtained.

###  Chemical characterization of hydrogels by Fourier transform infrared spectroscopy (FTIR)

 FTIR (FTIR, ATR-FTIR Thermo Nicolet model: NEXUS 670, USA) was performed in the wavenumber range of 3500-500 cm^-1^ with the scan rate of 4 cm^−1^ to identify chemical functional groups, bonding configurations, and molecular components.

###  Mechanical evaluation of hydrogels

 The wet hydrogels had been tested via the uniaxial compressive method by recruiting a Universal Testing Machine (SANTAM, STM-20, Iran). For this assessment, a round scaffold of every hydrogel group was prepared with the size of 1.5 and 2 cm as diameter and height, respectively. This device applied a force with the value and rate of 60 N and 2 mm/min, respectively. The assay had been carried out until the volume of the specimens reached 80% of the preliminary volumes. The force values were converted to stress by the below equation:


$$Peak\;stress\left(MPa\right)=\frac{F}{A}$$

Herein, F and A are respectively the ultimate loading force (Newton) and the cross-sectional area of the sample (m^2^).

 Moreover, the strain magnitudes were obtained according to the below formula:


$$Strain\;\left(\%\right)=\frac{L-L0}{L0}\times 100$$

Where, L0 is the initial height of the sample and L indicates the compressed dimension.

 In the end, the typical stress- strain curve was plotted and the slope of its linear region was reported as Young’s modulus (E).

###  Cell isolation and seeding on hydrogels 

 Both cell sources, including chondrocytes and human adipose mesenchymal stem cells (hMSCs), were derived from human tissues by the following protocols according to the ethical standards of Shahid Beheshti University of Medical Sciences (Tehran, Iran) after taking consent from healthy people. For the isolation of hMSCs, the adipose tissue was washed several times with phosphate buffer saline (PBS, Gibco) containing antifungal and antibacterial agents (amphotericin and pen/strep, Gibco) to remove oil droplets and blood residue on the tissue. The digestion process was started with collagenase I and IV (Gibco) at a concentration of 0.1%. The tissue was stored in an incubator for 2 hours and shaken every 15 minutes. The cells were then collected by centrifugation at 1200 rpm for 5 minutes. The enriched cells were suspended in high glucose Dulbecco’s modified Eagle’s medium (DMEM, Gibco) supplemented with 10% of fetal bovine serum (FBS, Gibco) and the noted antifungal and antibiotics. After 3 passages, the cells were counted and about 20 × 10^3^ cells were cultured per 1 cm of hydrogel. The cells were characterized by CD34, CD105, and CD45 fluorescent conjugated antibodies via flow cytometry technique (The data not shown). Moreover, prior to cartilage digestion, this tissue was cut into 1 mm^3^ pieces and treated with collagenase type II and IV (BioFroxx) at a concentration of 0.1%. The cells were incubated for 40 min and then, the resultant cells were cultured in cell culture media of DMEM supplemented with FBS. After that, the cells were seeded at 200 × 10^3^ cells per 1 cm^3^ and the medium was refreshed every 2 days.

###  Alkaline phosphatase (ALP) activity of cells loaded inside hydrogels

 Osteogenic regeneration was illustrated by measuring ALP activity. The assay was done after 14 days by the employment of an ALP kit (Pars Azmoun Co., Tehran, Iran). First, the total protein was extracted from the cells after their treatment with radioimmunoprecipitation assay lysis buffer (RIPA), and the digested cells were collected after centrifugation at 12 000 rpm for 10 minutes. P-nitrophenyl phosphate (5 mM) was employed for this assessment as a phosphatase substrate and after 1 hour at room temperature in dark conditions, the enzyme amount was obtained by reading their absorbance at 405 nm. The absorbance quantities were converted to the concentration by the standard curve of albumin (Sigma).

###  Alcian blue staining of cells loaded inside hydrogels 

 For characterization of cartilage differentiation, Alcian blue staining was done after 21 days. The cells of the hydrogel specimens were fixed with paraformaldehyde (Sigma, 4%) for at least 24 h and the cells had been then dried via ethanol gradients of 70%, 80%, 90%, and 100%. The hydrogels were located in paraffin and sectioned with the aid of a microtome device (Leica RM2155, Leica, Wetzlar, Germany). Then, the sections were deparaffinized and rehydrated to determine the density of sulfated glycosaminoglycan (GAG). Alcian blue stain (Merck) at 0.1% dilution in a solution of MgCl2 (0.4 M) and sodium acetate (pH 5.6, 0.025 M), was applied to the sections. At last, the tissue sections were mounted on the slides and examined by an optical microscope (KEYENCE, Osaka, Japan).

###  4 ′, 6-Diamidino-2-phenylin-dole (DAPI) and immunostaining procedures of cells loaded inside hydrogels 

 For the DAPI analysis method, the cells cultured within the scaffolds were washed with PBS after 21 days and then, DAPI (Sigma, 1 µg/mL) was added. The plates were incubated for 30 s and rinsed again with PBS to discard the unreacted DAPI stain. For the immunostaining method, Collagen I and II antibodies (Santa Cruz Biotechnology) were used to determine subchondral and chondral differentiation, respectively. The scaffold specimens were fixed after 21 days by incubation in paraformaldehyde (4%) for 2 hours at 4 °C. Goat serum (Gibco, 5%) solution was used for 1 hour to block non-specific epitopes. After using the primary non-conjugated antibodies, the second antibodies which were conjugated to fluorescein isothiocyanate (FITC), were delivered to the scaffolds. After all, a fluorescence microscope (Nikon, Eclipse TE2000-S, Japan) was recruited to observe the cell staining results in all hydrogel groups.

###  Statistical analysis

 Herein, Sigma plot software was used to see the difference between the data. The statistical tests including student’s t-test and one-way ANOVA were utilized to compare 2 and more than 2 groups. P-values less than 0.05 were considered a significant difference. It must be added that all values were expressed in this study as mean ± standard deviation (SD).

## Results and Discussion

###  Characterization of hydrogels by SEM 

**Figure 2:**
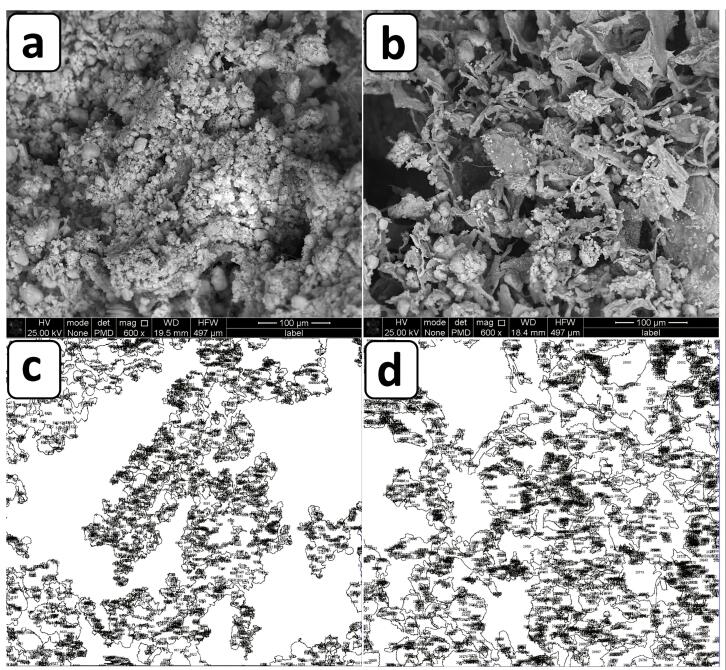
SEM evaluations of a) CEC-AHA hydrogel, b) alginate-nHAP hydrogel, c) porosity pattern of CEC-AHA hydrogel, d) porosity pattern of alginate-nHAP hydrogel. CEC and AHA are the abbreviations of N-Carboxyethyl Chitosan and aldehyde hyaluronic acid, respectively

**Figure 3:**
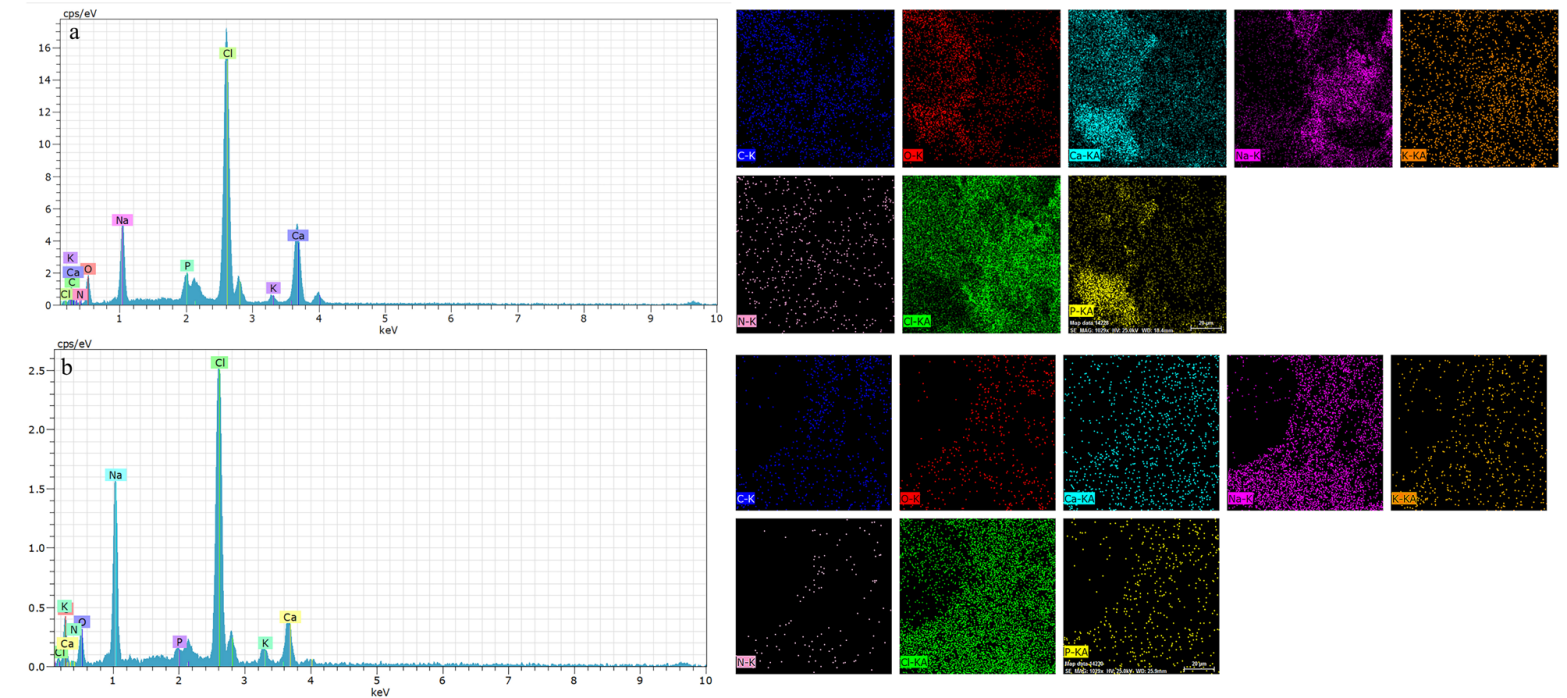
EDAX analysis of a) alginate-nHAP hydrogel and b) CEC-AHA hydrogel

 The micrographs of the hydrogels including osteogenic and chondrogenic lineages, were examined by SEM method. The results of this assay and also, their evaluations by ImageJ are shown in Figure 2. These observations confirmed a 3D sponge microstructure which is required for regenerative approaches of both tissue types. According to previous studies, the porous architecture could provide a suitable network for cellular migration and also nutrient transition.^17^ In spite of this, it should be added that this property plays a more efficient role, when the pores are interconnected to facilitate the diffusion of compounds including nutrients and waste materials. On the other hand, frameworks with a high degree of porosity indicate lower mechanical behavior.^18^ One study reported that the porosity level of scaffolds for cartilage tissue should be up to 71%^19^ to ensure sufficient mechanical strength of that scaffold for this tissue. Another investigation reported that scaffold with a porosity of higher than 70% can make a similar extracellular matrix (ECM) architecture to a normal type.^20^ The corresponding value is 60% for subchondral tissue.^21^ In the present study, the porosity values of the subchondral and chondral hydrogels were 61.45 ± 4.51 and 44.145 ± 2.81 %, respectively. The porosity of the osteogenic hydrogel was higher than that of the chondral type, supporting the significant effect of the crystalline nature of nHAP compared to the polymers. The smaller pore size of the chondral scaffold due to nHAP has been approved by others. The main reason for this phenomenon is related to more ice crystal nucleation sites in the presence of nHAP.^22^ On the other hand, the pore size seems to be smaller and more homogenous with the subchondral layer and nHAP was distributed uniformly. The nanoparticles are small enough not to disturb the hydrogel structure. In contrast, the type of chondral hydrogel based on two different polymers, including CEC and AHA, changes significantly the diameters of the pores. To the best of our knowledge, unlike minerals with blocky shapes, polymer chains are rather space-filling due to their long linear structures. In particular, it has been approved that the alginate hydrogel always has a sponge-like structure, and in accordance with other observations, its pore size may reach to 100-150 µm.^23^ Here the pore size of the alginate reached 68 ± 15 µm in the presence of nHAP. Although, the optimal pore size for bone regeneration has been reported to be 300-500 µm for better collagen production, HAP deposition, and osteoblast maturation.^24^ Compared to other examinations, it could be concluded that the pore size of the alginate composite scaffold is relatively small, which is related to the effective role of nHAP during the lyophilization process. These contradicting data about the pore diameters could be related to the complexity of the 3D culture for cell migration and nutrient transfer. On the other hand, other examinations recommended that larger pore diameters promote chondrocyte proliferation and matrix development.^25^ In this manner, the pore diameter of 156 ± 70 µm for chondral repair approaches, can develop greater production of collagen II and glycosaminoglycan. In addition, the elemental composition of the subchondral hydrogel by EDAX confirmed that the main normalized weights belonged to O, C, Cl, Ca, and P as 25.50%, 20.14%, 25.41%, 13.27% and 2.5%, respectively. These elements originated from alginate, CaCl_2_, and nHAP. In this manner, the respective large weights of the chondral layer resulted for O, C, Cl, Na, and N as 20.20%, 36.86%, 18.60%, 13.55%, and 4.16%. In accordance with the compounds which were used for the fabrication of this scaffold, these peaks are representative of CEC, AHA and sodium periodate. However, the presence of Cl with this scaffold could be related to the presence of some impurities with these polymers (Figure 3).

###  Chemical characterization by FTIR analysis

**Figure 4:**
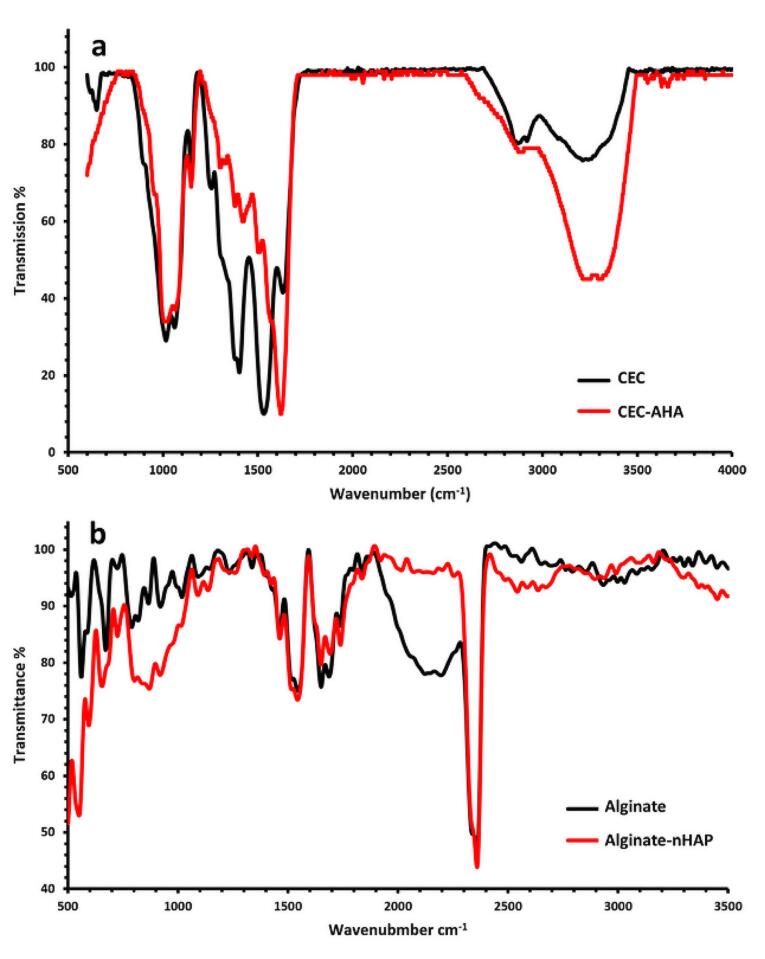
FTIR spectra of a) the CEC and CEC-AHA hydrogels and b) FTIR spectra of the alginate and alginate-nHAP hydrogels. CEC and AHA are the abbreviations of N-Carboxyethyl Chitosan and aldehyde hyaluronic acid, respectively

 FTIR analysis was examined to find functional chemical groups of the alginate-nHAP and CEC-AHA. In this manner, Figure 4 compares the FTIR spectroscopy of the Alginate-nHAP and CEC-AHA with the peaks of the alginate and CEC, respectively. The important bands of chitosan including C = O and NH2 appeared due to their stretching vibrations at 1624 cm^−1^ for carbonyl^26^ and 1539 cm^−1^ for amine.^27^ The higher intensity of the C = O band after the reaction of CEC and AHA, confirmed the covalent bonds between these two polymers. In other words, as a result of the hydrogel preparation, the amount of carbonyl groups was increased due to the reaction of carboxyl and aldehyde derivatives of CEC and AHA, respectively. The aromatic bands at 650 cm^−1^ belong to the saccharide rings of chitosan in the CEC hydrogel, while this band disappeared after the hybridization of CEC with AHA.^28^ The peaks of AHA were at 1565^29^ cm^−1^ and 1315^30^ cm^−1^ representing amide II and III. A very small peak is detected in the chitosan group at 3257 cm^-1^ as a function of N-H bending vibrations,^31^ which intensifies after its blending with AHA. The asymmetric bridge oxygen of C-O-C at 1157^32^ and 1022^33^ cm^−1^, is exposed with the hybrid scaffold. On the other hand, the sp^2^ C–H stretches occurred at 3090 cm^−1^.^34^ Moreover, a considerable peak at 1250 cm^−1^ is apparently assigned to C-N bonds^35^ with the CEC-AHA scaffold. In the subchondral scaffold, the wavenumbers of 551 and 599 cm^−1^ are representative of P-O groups with nHAP^36^ and other phosphate stretches were observed at 1098 cm^−1^.^37^ The characteristic bands of the carbonate groups were exposed at 871 and 1461 cm^−1^.^38,39^ C-O stretching of the pyranosyl rings with alginate, attenuated the peaks of 921^40^ and 1018 cm^−1^.^41^ The corresponding disappearance may be related due to the interactions of the phosphate and carbonate groups of nHAP. The carboxylic functional groups of alginate were detected at 1422 cm^−1^.^42^ These peaks persisted even after the interactions of alginate with nHAP.

###  Characterization of hydrogels by compressive methods

**Figure 5:**
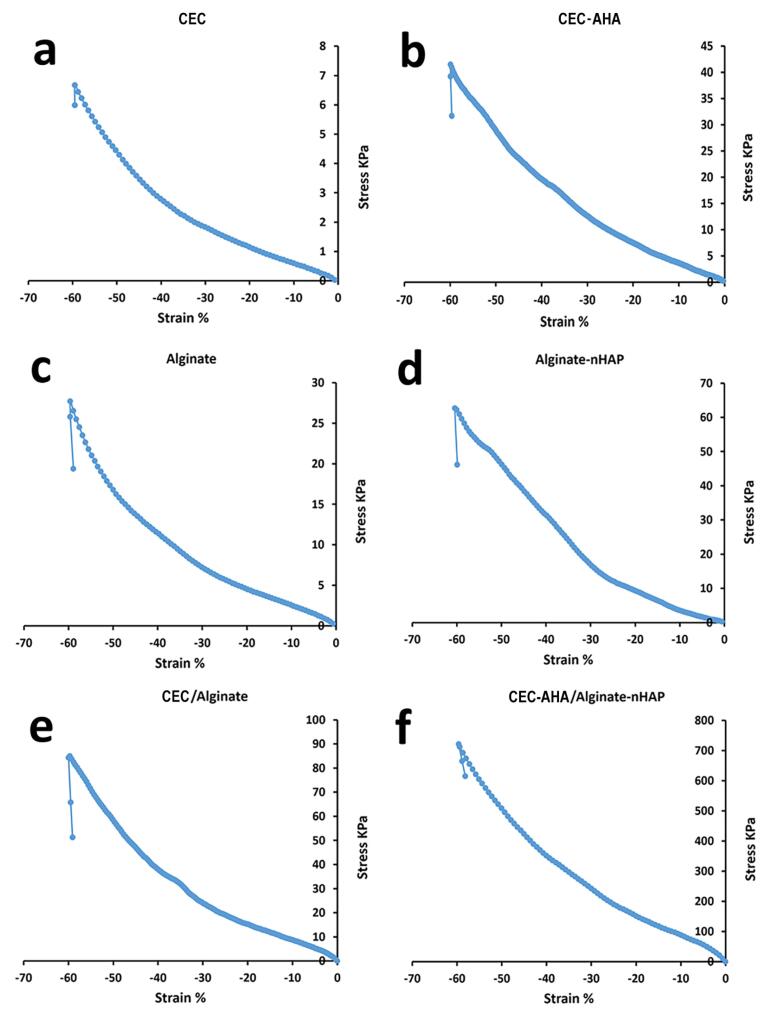
Compressive properties of a) CEC hydrogel, b) CEC-AHA hydrogel, c) alginate hydrogel, d) alginate-nHAP hydrogel, e) CEC/alginate hydrogel and f) CEC-AHA/alginate-nHAP hydrogel. CEC and AHA are the abbreviations of N-Carboxyethyl Chitosan and aldehyde hyaluronic acid, respectively

 The prepared hydrogels including CEC, CEC-AHA, alginate, and alginate-nHAP, and also, their pure and composite biphasic types, were evaluated by compressive assay. The results are summarized in Figure 5 and Table 1. First of all, it should be noted that hydrogels cannot be expected to have high mechanical strength due to their high water capacity.^43^ In accordance with the corresponding accompanying data, when the CEC polymer was cross-linked with AHA, Young’s modulus was increased from 6.07 ± 0.23 to 46.39 ± 6.51 kPa. Also, their ultimate strength and elongation were increased from 6.67 ± 0.71 to 41.45 ± 3.64 kPa and 59.5 ± 3.32 to 59.89 ± 4.42 %, respectively. However, the difference between their strain (%) was not statistically considerable (*P* value > 0.05). The higher mechanical properties of CEC-AHA were correlated with the formation of tight ionic bonds between these polymers. It agrees with the results of a study that investigated the influence of the HA coating on the mechanical properties of chitosan fibers.^44^ While another group reported lower mechanical properties after adding HA to chitosan.^45^ This contradiction could be due to the different chitosan/HA ratios and also, their various cross-linking procedures. Nevertheless, the mechanical characteristics of the hydrogel resulting from the cross-linking in this study, justify its endurance compared to the pure CEC. Thereby, the CEC hydrogel lacks mechanical strength versus its hybrid hydrogel with AHA. The subchondral hydrogels were divided into the alginate and alginate-nHAP types as the control and test groups, respectively. Apart from the stretching property (*P* value > 0.05), the other values related to the elastic modulus and the final resistance against the loading force, triggered from 22.98 ± 3.36 to 35.15 ± 5.24 and 27.67 ± 2.81 to 62.65 ± 4.11 kPa, respectively (*P* value < 0.05). These observations were consistent with other reports confirming a higher stiffness value with the composite scaffold compared to the bare type.^46^ In one investigation, it was discussed that this result is related to the enhancing effect of nanoparticles. In this manner, the polymer chains transfer the force to nHAP and these particles are pulled out along with the applied force and thus, progressing strength resistance.^47^ It is interesting to notice that when comparing the biphasic hydrogels, including the composite and bare types, the strain value (%) was essentially the same (*P* value > 0.05). This feature is definitely due to their same polymer concentrations. While both modulus and maximum strength were repeatedly increased from 76.38 ± 4.41 to 108.33 ± 5.56 and 84.91 ± 3.38 to 721.135 ± 8.21 kPa, respectively for the bare and composite hydrogels. In accordance with the above explanation, these higher values could be justified by the presence of AHA and nHAP with the composite scaffold. According to other reports, the modulus of human cartilage is between 500 and 1000 kPa.^48^ Nevertheless, the composite biphasic hydrogel prepared in the present study, demonstrated a compressive modulus value of 108.33 ± 5.56 kPa, which is less than the target level for human use. However, the maximum force that this scaffold can withstand is 721.135 ± 8.21 kPa, which confirms its stability to high load power. In addition, considering the strain value (59.60 ± 7.23 %), this hydrogel shows elastic behavior and sufficient flexibility of more than 50% of the original length. Therefore, this hydrogel can be highly compressed and return to its initial size without breaking.^49^ Regarding to the concept of subchondral mimicry, the mechanical functionality of the prepared alginate-nHAP scaffold is obviously not comparable to the mechanical capacity of normal subchondral bone tissue, which has Young’s modulus in the range of 297 MPa to 20 GPa.^50^ In spite of this, its behavior might be prominent in some studies when comparing the mechanical properties of this biphasic composite scaffold with other hydrogels. In one study, a scaffold made from hydroxybutyl chitosan-oxidized chondroitin sulfate, showed a maximum strength of 7 kPa and the same elongation value as our biphasic composite group.^51^ Although it is necessary that the fabrication of this bilayer scaffold is not only performed in the presence of these natural polymers, a synthetic polymer should also be added to gain an optimal scaffold with more mechanical similarities to normal tissue.^52^

**Table 1:** The compressive properties of the different scaffold groups are shortened. CEC and AHA are the abbreviations of N-Carboxyethyl Chitosan and aldehyde hyaluronic acid, respectively

**Scaffold type/Mechanical property **	**Young modulus (kPa)**	**Maximum force (kPa)**	**Maximum elongation (%)**
CHC	6.07 ± 0.23	6.67 ± 0.71	59.5 ± 3.32
CHC-AHA	46.39 ± 6.51	41.45 ± 3.64	59.89 ± 4.42
Alginate	22.98 ± 3.36	27.67 ± 2.81	59.65 ± 6.81
Alginate-nHAP	35.15 ± 5.24	62.65 ± 4.11	60.46 ± 7.12
CHC/Alginate	76.38 ± 4.41	84.91 ± 3.38	59.67 ± 1.56
CHC-AHA/Alginate-nHAP	108.33 ± 5.56	721.135 ± 8.21	59.60 ± 7.23

###  Characterization of cells loaded inside monophasic hydrogels by SEM 

**Figure 6:**
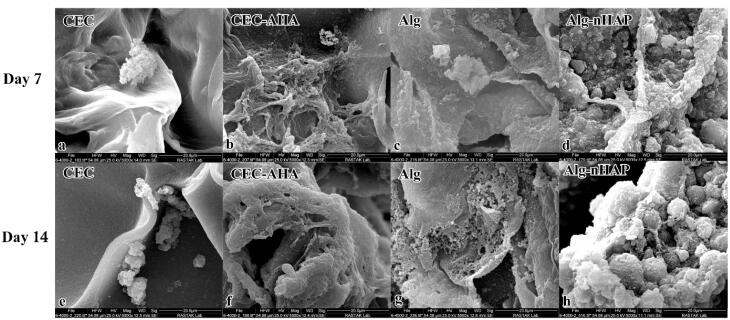
SEM evaluations of cells cultured in a) CEC hydrogel after 7 days, b) CEC-AHA hydrogel after 7 days, c) alginate hydrogel after 7 days, d) alginate-nHAP hydrogel after 7 days, e) CEC hydrogel after 14 days, f) CEC-AHA hydrogel after 14 days, g) alginate hydrogel after 14 days and h) alginate-nHAP hydrogel after 14 days. CEC and AHA are the abbreviations of N-Carboxyethyl Chitosan and aldehyde hyaluronic acid, respectively

 The morphology of chondrocytes and hMSCs on the hydrogels was evaluated by SEM after 7 and 14 days of the cell seeding. In accordance with other studies, hMSCs are generally 15-50 µm in size with a mean diameter of 26.5 ± 0.4 µm.^53^ In the present study, the SEM examinations (Figure 6) revealed the diameter of their spheroids was 16 ± 2.4 µm after 7 days and this value increased to 23 ± 1.7 µm at 14 days (*P *value < 0.05). In this regard, the diameter along their expansion was 36 ± 3.3 µm which increased to 43 ± 5.1 µm when they were cultured in the presence of nHAP. However, the surface of these spheroids was obviously covered by the minerals secreted by the osteogenic cells. It is clear that not only the amount of these inorganic deposits increased over time, but their size also enhanced with the composite scaffold in the presence of nHAP. In addition, it should be added that the phenotype of the spheroids with the composite group compared to the bare one, was more linear and they lost their fibroblast-like spindle shape. Owing to the hydrophilic character of HAP through its hydroxyl groups, it was expected that there would be no limitation on cell adhesion and spreading in the presence of HAP. However, an investigation approved that cells show less spread and maintain a round configuration than the group without HAP coating.^54^ This observation contradicts the result of the present study. On the other hand, there are some studies that cells elongated and well-spread with narrow cellular extensions in the presence of HAP,^55^ These contradictory results could be related to the use of different polymers as matrix elements. In addition, the size of chondrocytes changes over time, since when cultured on a hydrogel, their diameter was 20 µm after 7 days and this size reached 40 µm after 14 days. Although, this scale relates to their culture in a medium supplemented with L-ascorbic acid-2- phosphate.^56^ In the absence of a chondrogenic factor, the size of these cells is expected to indicate lower conversions. Considering this truth, the loaded cells within the bare chondral hydrogel had some spheroids with a size of 15 ± 1.5 µm after 7 days that increased to 17 ± 2.1 µm after 14 days (*P *value < 0.05). While, when the cells were seeded inside the hybrid network, the diameter of the spheroids was measured as 22 ± 3.1 µm at 7^th^ days of the culture, which widened to 35 ± 1.1 µm (*P *value < 0.05) after 14 days. Moreover, it is clear there are many lacunae in the control group compared to the CEC-AHA hydrogel, representing that the number of chondrocytes was insufficient to occupy the entire area. In contrast, hyaluronan due to its mitotic effect on chondrocytes,^57^ can increase cell population and hence, cell colony size was improved with the hybrid group. Overall, the corresponding cells in the CEC-AHA hydrogel, were able to expand better and the AHA component promoted their morphology into the cells with the ability to develop ECM.

###  Characterization of cells loaded inside biphasic hydrogels by SEM 

**Figure 7:**
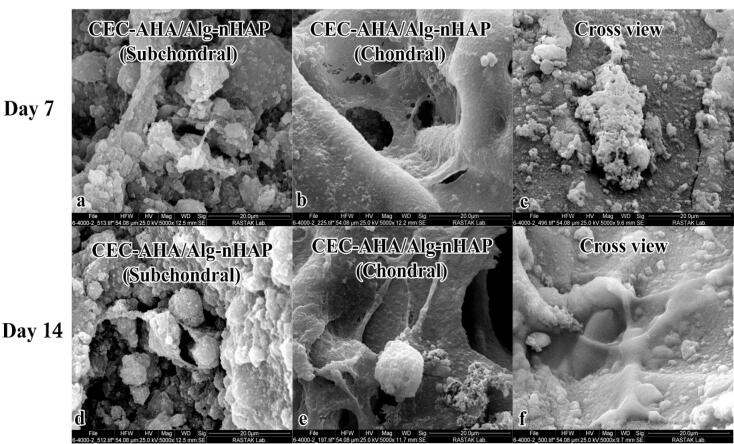
SEM evaluations of cells cultured in a) CEC-AHA/alginate-nHAP hydrogel after 7 days formed the subchondral phase, b) CEC-AHA/alginate-nHAP hydrogel after 7 days formed the chondral phase, c) CEC-AHA/alginate-nHAP hydrogel after 7 days form cross -sectional view, d) CEC-AHA/alginate-nHAP hydrogel after 14 days formed the subchondral phase, e) CEC-AHA/alginate-nHAP hydrogel after 14 days forms chondral phase and f) CEC-AHA/alginate-nHAP hydrogel after 14 days as the cross-sectional view. CEC and AHA are the abbreviations of N-Carboxyethyl Chitosan and aldehyde hyaluronic acid, respectively

 The outcomes in Figure 7 depicted the biphasic hydrogels including CEC-AHA/alginate-nHAP from the subchondral, chondral, and cross-sectional views. The cellular spheroids are evident in all groups showing no intervening relationships between chondrocytes and hMSCs. The morphology and size of the cell clusters are consistent with the results of the monophasic hydrogels. The osteogenic cell mass on the dual composite scaffold had a mean diameter of 12 ± 6.2 after 7 days, which was completely covered with minerals after 14 days. The chondral cells proliferated vigorously and their flattened phenotype filled the entire space at both 7 and 14 days, although the cellular spheroid formation occurred after 14 days. By consideration of the cross-sectional view, an increasing rate of cell count was detectable from 7 to 14 days. This evidence confirmed that the biphasic co-cultured condition could help to improve osteogenic and chondrogenic differentiation. The result is in agreement with a study about the positive supportive role of hMSCs on chondrocytes, due to their greater resemblance to the niche *in vivo.*^58^ On the opposite, it has been found that when chondrocytes are cultured alone, their polygonal phenotype changes to round or flattened over time.^59^ It has even been approved that some hMSCs differentiate into chondrocytes and start producing collagen II.^60^ Also, it has been explored that the co-cultured strategy inhibits chondrocyte calcification.^61^ Another examination depicted that hMSCs showed no contribution to proteoglycan deposition with chondrocytes under a co-culture condition, except in the presence of TGF-β1 and dexamethasone as biochemical factors. Moreover, ALP expression was increased by co-culture in the presence of these small biomolecules.^62^ In view of this study, it can be concluded that AHA and nHAP act as triggers to both chondral and subchondral lineages. As a result, these co-cultured cells exhibit a paracrine effect on each other through growth factor secretion as well as cellular interactions. However, the co-cultivation system is a well-known strategy to meet signal requirements and simulate niche conditions. In particular, this process appears to be a facilitating factor for chondral regeneration due to paracrine signaling from hMSCs on chondrocytes. One study confirmed the high proliferation of chondrocytes in the presence of MSCs and the longer maintenance of their cartilaginous phenotype as a function of growth factors secreted by MSCs.^60^ Furthermore, it was confirmed that some MSCs gained the potency to become chondral cells under a co-harvesting condition with chondrocyte.^63^ Therefore, there are some expectations that the fate of MSCs in this group is doomed to differentiate into chondral cells. In this regard, although HAP is an osteogenic component, the potency of these cells to osteogenic cell type would be reduced to some extent. Other studies, albeit with less evidence support this hypothesis that in osteochondral tissue engineering, there is a challenge in the chondral phase and not in the subchondral one. A study approved that a scaffold containing HAP, can successfully integrate into the host bone and reconstruct the subchondral layer in contrast to the chondral part.^64^ Overall, there is no concern about the osteogenic conversion of MSCs due to HAP and even the cartilage layer would be strengthened under the co-culture condition of MSCs and chondrocytes.

###  Characterization of cell differentiation by ALP activity

**Figure 8:**
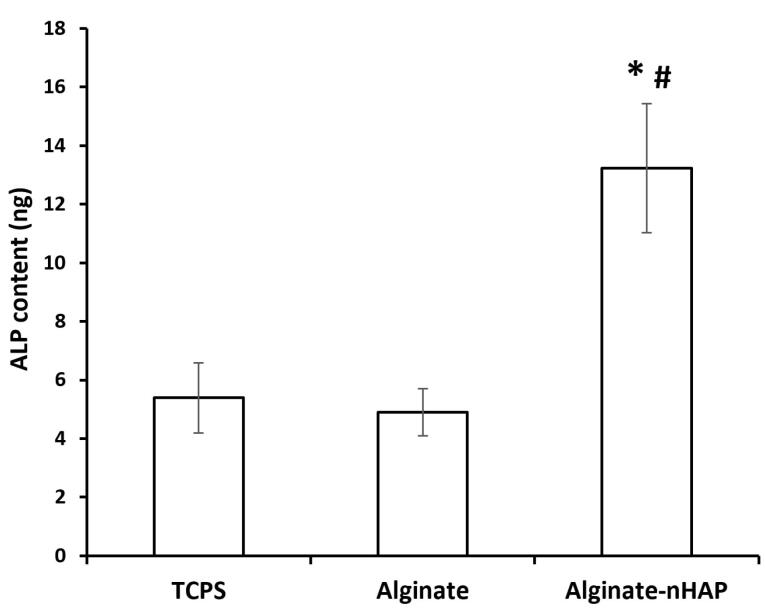
ALP content of TCPS, alginate and alginate-nHAP after 14 days was plotted

 The osteo-promoting function of the alginate-nHAP scaffold on hMSCs was evaluated via the ALP method. This assessment could measure osteogenesis *in vitro* and confirm the osteogenic potency of this scaffold *in vivo.*^65^ In this manner, after 14 days, the osteogenic differentiation of hMSCs was investigated between the alginate and alginate-nHAP. The ALP gene has been mostly reported to be an early marker during osteogenic differentiation.^66^ Therefore, according to other surveys, the reasonable time to assess its expression is less than 14 days.^67,68^ The results supported the considerable effect of nHAP on the expression of this enzyme as previously reported.^69^ It is clear from Figure 8 that there was a distinguishable relation (*P* value < 0.05) between the composite groups (13.3 ± 2.2 ng) compared to the tissue culture polystyrene (TCPS, 5.39 ± 1.2 ng) and alginate hydrogel (4.9 ± 0.8 ng). It should be added that the osteoinductive effect of this nanoparticle type is related to the activation of the Wnt/β-catenin signaling pathway.^70^ In contrast to the composite scaffold, the bare Alg and TCPS groups were relatively equal (*P* value > 0.05) and even, the TCPS group had a higher osteogenic potential compared to the bare hydrogel group. Consistent with other literature, the mechanism of osteoclasts on bone resorption has been clearly explained, however scaffold decomposition as *in vitro* and *in vivo* models is still relatively unknown. The process described for HAP degradation is the hydrolysis pathway.^71^ During HAP decomposition, Ca and P ions released into the environment play an effective role in cells to differentiate osteogenic lineage. It is evident that in the alginate and TCPS groups, due to the lack of nHAP, this osteogenic commitment is not to be expected. It should be noted that HAP can trigger cell proliferation^72^ and this consequent mitotic impact, drives cells to a high cell population and ultimately increases mineral deposition.

###  Characterization of cell differentiation by Alcian blue

**Figure 9:**
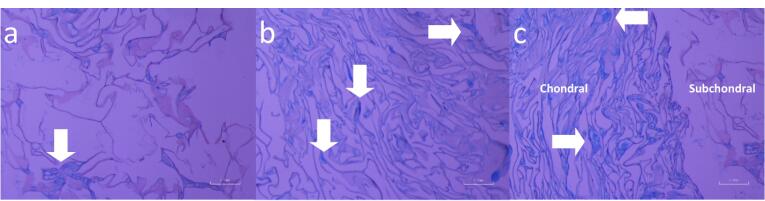
Alcian blue staining of a) CEC hydrogel, b) CEC-AHA and c) CEA-AHA/alginate-nHAP. The white arrows indicate the proteoglycans which were colored by Alcian blue stain. CEC and AHA are the abbreviations of N-Carboxyethyl Chitosan and aldehyde hyaluronic acid, respectively

 Alcian blue staining was also established in the present study to confirm chondral differentiation colorimetrically. GAGs are the main components of the chondral-specific matrix and with a larger amount of this compound, the blue stains would be numerously generated as a result of reactions between Alcian and GAGs. As it is apparent from Figure 9, proteoglycan-rich scaffolds could be observed in both the chondral (CEC-AHA) and dual chondral-osteogenic scaffolds (CEC-AHA/alginate-nHAP). However, the CEC hydrogel which was not cross-linked by AHA, indicated a lower level of proteoglycan-positive regions. The cell type seeded in all these scaffolds, was chondrocytes and therefore, Alcian blue staining is expected for them. Although, a scaffold with better conditions for cells to maintain the expression of marker genes and ECM secretion, would be a better candidate for chondral regeneration. According to the related data, the CEC hydrogel as the AHA-free group indicated bright and strong blue color with a sparse population, while the group containing the AHA component, possessed strong positive regions of Alcian stain with high frequency. In agreement with other studies, the production of proteoglycans such as chondroitin sulfate is triggered in the presence of the hyaluronan derivative.^73^ Also, it should be added that chitosan may have a chondrogenic effect to some extent due to its proteoglycans-like structure,^74^ although its corresponding capacity could be enhanced in the presence of specific chondral media conditions. It is crucial for proper chondral repair that chondrocytes maintain their ability to develop a cartilage-specific matrix. Herein, the positive results of blue staining, support that the cultured chondrocytes stored their relevant functional potency for GAG generation. Therefore, it can be concluded that hyaluronan inhibits the senescence process of chondrocytes. This phenomenon was reported in a study that hyaluronan oligosaccharide induces the formation of MMP-13, which breakdowns the chondral matrix. The interactions of hyaluronan fragments with CD44 and CD54 inhibit chondrocyte apoptosis.^75^ On the other hand, hyaluronan has a positive effect on chondrocyte proliferation,^57^ which distinguishes the AHA-rich hydrogels from the groups without this polymer. However, there was a distinguishable boundary between the chondral and subchondral layers in Figure 9c, some chondrocytes migrated to the osteogenic phase and thereby, produced some proteoglycans on the other side. The migration of these cells has been confirmed by other reports demonstrating that if successful integration between cartilage and other tissues occurs, a neo-ECM would be generated at the interface of both tissues.^76^ This integration guarantees the creation of a mechanically resistant organ in the future.

###  Characterization of cell differentiation by immunostaining methods

**Figure 10:**
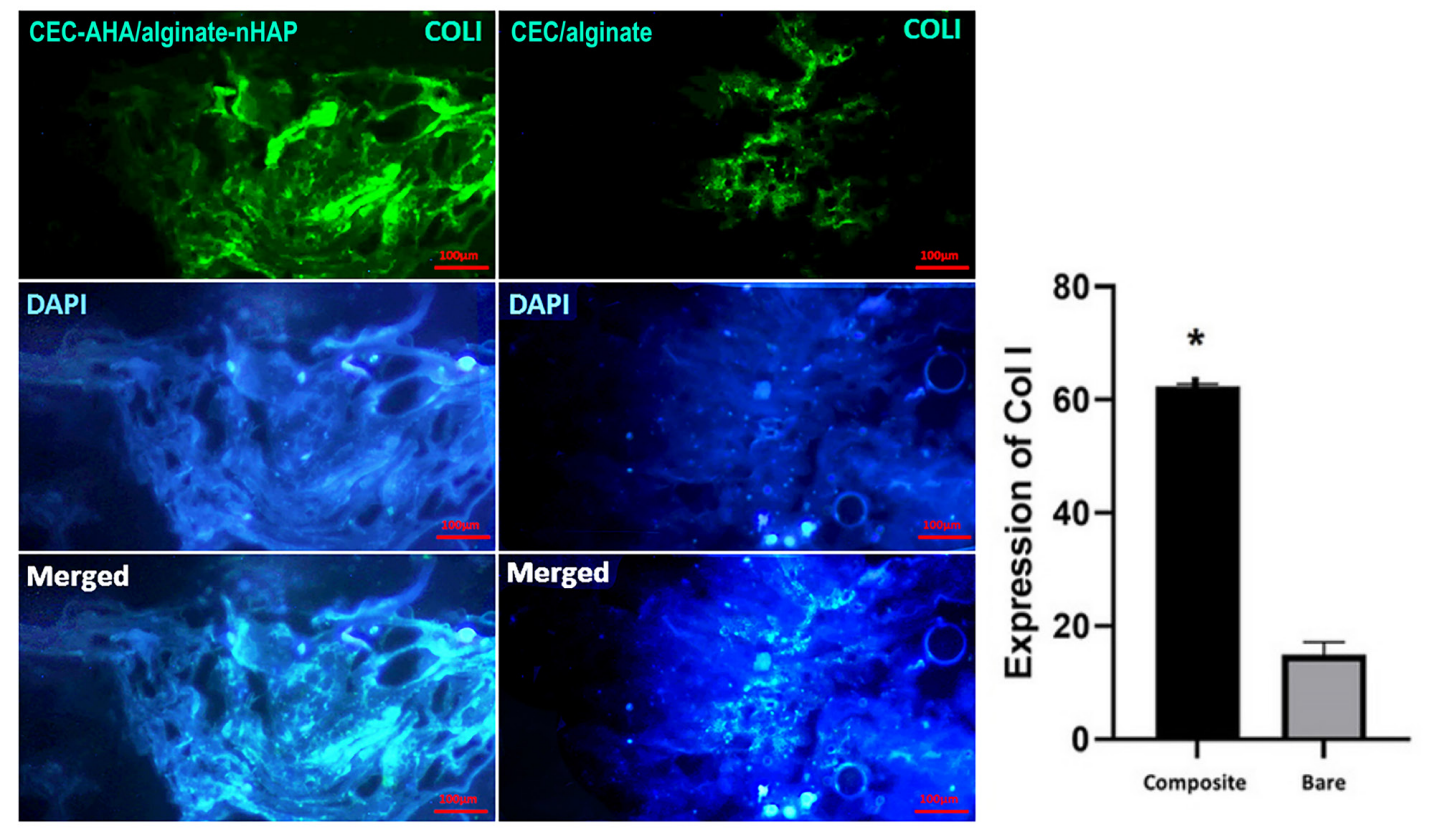
Collagen I immunostaining of CEC-AHA/alginate-nHAP and CEC/alginate hydrogels. The scale bar is 50 µm. CEC and AHA are the abbreviations of N-Carboxyethyl Chitosan and aldehyde hyaluronic acid, respectively

**Figure 11:**
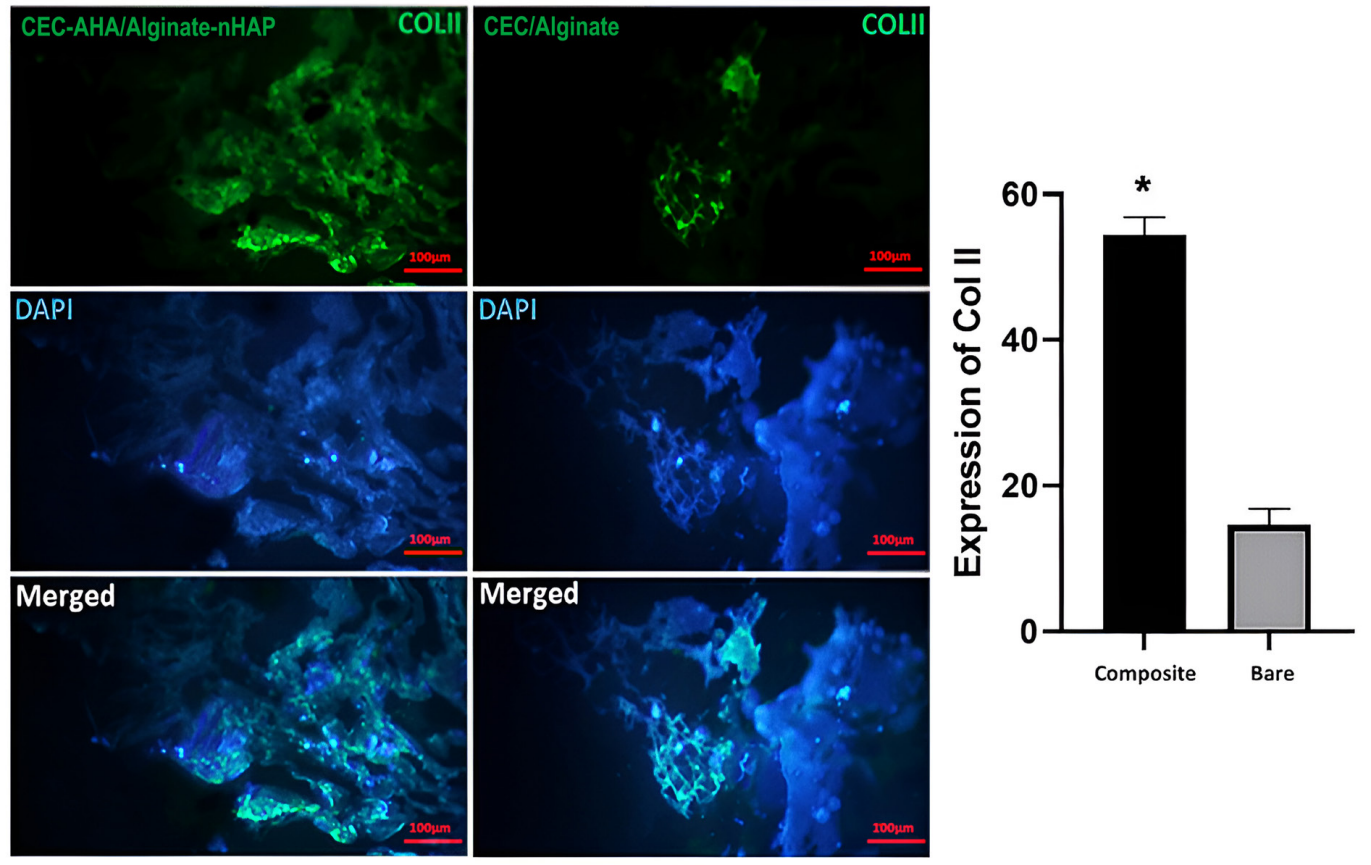
Collagen II immunostaining of CEC-AHA/alginate-nHAP and CEC/alginate hydrogels. The scale bar is 50 µm. CEC and AHA are the abbreviations of N-Carboxyethyl Chitosan and aldehyde hyaluronic acid, respectively.

 Generation of ECM specific to osteogenic and chondral type, was characterized by the expression of collagen I and II proteins, respectively. Type I collagen confirmed early osteogenic mineralization^77^ and is well-known as the major protein of bone tissue. On the other hand, the second type of this protein is specified to the ECM of cartilage tissue, which consists predominantly of the ECM of this tissue.^78^ In this paper, the expression of these proteins between the bilayer scaffolds with and without nHAP and AHA components, was compared. From Figures 10 and 11, it can be seen that the highest osteogenic and chondrogenic induction occurred in the scaffolds, which was accompanied by HAP and AHA. The relationships between both scaffold types are statistically considerable (*P* value < 0.05) approving the beneficial effects of nHAP and AHA. The respective pixel values of composite and bare scaffolds for collagen I were 62 ± 1.7 and 17 ± 2.5. Regarding to collagen II, the corresponding amounts were 54 ± 3.8 and 12 ± 4.1. The calcification of hMSCs by nHAP, had been reported in previous studies due to its involvement in the induction of some sequential genes including integrins, proliferative genes, and also ALP.^14^ It has been found that bone apatite induces high migration, differentiation, and mineral deposition of osteoblasts. Its mechanism is relevant to the activation of integrin binding sialoprotein (IBSP) and dentin matrix protein 1 (DMP1). The higher expression of these proliferative genes, causes a pronounced production of collagen I by cells, as shown in this study. One group reported that HAP can stimulate a long-term osteogenic situation for 42 days.^79^ In addition to these pathways, HAP platelets augment the expression of type I collagen, and its mechanism is related to better cellular activity in the presence of HAP and consequently, greater collagen synthesis by cells. In the same way, the hyaluronan component provides a hydrated space and facilitates cell proliferation,^80^ repeatedly leading to the greater expression of collagen II. Moreover, chondrocytes interact with the hyaluronan matrix through CD44 on their membrane and this phenomenon promotes the formation of cartilage matrix.^81^ The weak expression of these marker proteins by the bare bilayer scaffolds demonstrated that alginate and chitosan polymers can weakly induce progenitor or stem cell differentiation. While it has been recognized that the production of collagen by cells is concentrated in the regions of chitosan abundance. Despite this fact, it should be added that if the chitosan scaffold had sufficient porosity for cell penetration, the cells would migrate deeply and collagen formation would not be restricted to the surface of the chitosan scaffold but to its interior. Considering the results, based on the presence of the stains throughout the scaffold area with the composite scaffold, we concluded that there was a higher cell migration compared to the bare scaffold. Moreover, there is a saturation point for collagen production that has been quantitatively estimated by others to be between 11 and 14 days,^82^ and by considering the time point of this study (after 21 days), both types of scaffold had adequate time to become saturated. Accordance to other research, alginate could induce the expression of chondral marker genes such as aggrecan, collagen II, and SRY-box transcription factor 9^83^ or even, the osteogenic pathways.^84^ This process is verified in this study for chitosan as the base polymer of the chondral scaffold. It has been found that chitosan increases the mineral deposition of hMSCs.^85^ However, this polymer activates the chondrogenic pathway either in the presence of its condition culture medium^86^ or other parameters such as scaffold porosity. Taken together, these observations correlate with the fact that the cell commitment to both tissue types could happen simultaneously and the prepared biphasic scaffold could be chosen as a new strategy to resolve OA lesions.

## Conclusion

 Due to the effective role of nHAP and AHA in subchondral and chondral regeneration, the bilayer scaffold could be introduced as the base material for the fabrication of joint replacement implants. It should be noted that both cell sources can be obtained autologously and thereby, there are no concerns about immune system reactions. The small pore diameter of the osteogenic hydrogel can limit cell penetration, which is a critical parameter for integrating a scaffold with host tissue. In spite of this, the subchondral layer successfully supported cell transfer obtained from the cross-sectional view of the biphasic scaffold via SEM. This microstructure determines the mechanical strength of the scaffolds including the CEC-AHA and alginate-nHAP. The bone layer scaffold as a function of its small pores and also, the presence of nHAP as a brittle compound, had a lower Young’s modulus of 35.15 ± 5.24 kPa. Whereas the maximum strength of this phase was higher than that of the chondral layer, supporting the fact that powdered materials like HAP, can provide high strength under large compaction force. In terms of cell culture within the hydrogel contained nHAP, the cells were well elongated with cellular extensions. On the other hand, hyaluronan as a mitotic factor on chondrocytes, stimulated cell proliferation, and the developed cells occupied the whole area of the scaffold. The CEC-AHA scaffold demonstrated significantly greater Alcian blue compared to the alginate-nHAP layer in the biphasic composite scaffold. In this manner, it could be ensured that the nHAP loaded into the subchondral layer, activated osteogenic capacity only at this phase. However, some blue regions were exposed on this side, but it should be added that the population of these spots was very low. This satisfactory result was obtained with the biphasic group, especially in the chondral part. However, the ability of this scaffold requires to be evaluated by *in vivo* examinations and its effect on the expression of other ECM components should be monitored.

## Competing Interests

 There is no known conflict of interests between the authors that could impact on the results of the corresponding study.

## Ethical Approval

 The present study was approved by Shahid Beheshti University of Medical Sciences (the ethical number: IR.SBMU.RETECH.REC.1398.127).

## Funding

 This study was supported by Shahid Beheshti University of Medical Sciences(grant ID: 12550).
